# A vision-based framework for quantifying fish feeding behavior in industrial recirculating aquaculture systems

**DOI:** 10.1038/s41598-026-54934-x

**Published:** 2026-05-26

**Authors:** Changrui Hu, Ziquan Feng, Yuanhang Li, Juan Chen, Shanzhou Wei, Xin Guo, Zemin Zhang

**Affiliations:** 1 School of Artificial Intelligence Technology, Guangxi Technological College of Machinery and Electricity, No. 101 Da Xue Dong Road, Nanning, 530007 China; 2 School of Green Building and Low Carbon Technology, Guangxi Technological College of Machinery and Electricity, No. 101 Da Xue Dong Road, Nanning, China; 3 School of European and American Languages and Cultures, Guangxi University of Foreign Languages, No. 22 Zheng He Road, Nanning, 530222 China

**Keywords:** Recirculating aquaculture system(RAS), Feeding behavior analysis, Intelligent feeding, Computer vision, Ecology, Ecology, Environmental sciences, Ocean sciences

## Abstract

Accurate quantification of fish feeding intensity is critical for optimizing feeding strategies and reducing feed waste in industrial recirculating aquaculture systems (RAS). However, real-world aquaculture environments present significant challenges, including high-density fish populations, water surface disturbances, and dynamic behavioral variations. To address these issues, this study proposes a hybrid vision-based framework (HVIT) for robust feeding intensity analysis. The proposed method integrates a Convolutional Neural Network (CNN) for local feature extraction and a Vision Transformer (ViT) for global context modeling within a parallel architecture, enabling effective representation of complex group behaviors. Furthermore, a Long Short-Term Memory (LSTM) module is incorporated to capture temporal dynamics of feeding activity, allowing continuous characterization of feeding intensity over time. A dedicated dataset of largemouth bass (Micropterus salmoides) under industrial RAS conditions was constructed, with data augmentation strategies applied to improve robustness against environmental noise and visual disturbances. Experimental results demonstrate that the proposed framework achieves over 98% accuracy across four feeding intensity levels and outperforms conventional CNN-based approaches. More importantly, the proposed method enables quantitative evaluation of feeding activity, providing a practical basis for real-time feeding decision support and intelligent feeding system development in large-scale aquaculture.

## Introduction

In industrial RAS, feed constitutes the primary operational expenditure, often exceeding 50% of total production costs^2,11^. Empirical research consistently indicates that feeding intensity serves as a high-fidelity proxy for appetite^1,21,25,35^, thereby providing indispensable metrics for precision aquaculture management.

To quantify and analyze the foraging behavior of farmed teleosts, several monitoring methodologies have been developed. Current technologies for data acquisition primarily utilize diverse sensor arrays and computer vision systems. For example, acoustic sensors are deployed to evaluate both feeding intensity and consumption rates. The principal advantage of acoustic monitoring is its independence from ambient lighting conditions and its operational resilience in turbid underwater environments. However, the widespread commercial adoption of acoustic systems is frequently constrained by prohibitive costs and logistical complexities in signal processing. As an alternative, accelerometers may be integrated into the system to detect shifts in swimming kinematics and velocity during active foraging periods.

Extensive research has investigated teleost feeding behavior to optimize aquaculture practices. By analyzing salmon feeding data, Eriksen et al.,^7^ categorized feeding intensity into four distinct levels: Level 1 denotes an absence of feeding activity; Level 2 involves localized consumption of immediate feed; Level 3 represents active foraging followed by a return to the initial position; and Level 4 indicates aggressive feeding, characterized by the rapid consumption of all dispersed feed and significant surface splashing. Zhou,^34^ evaluated fish school aggregation dynamics and subsequently introduced the FIFFB index to detect feeding intensity, demonstrating high efficacy. Lee,^14^ applied automated individual counting and recognition algorithms to indirectly estimate concurrent feeding intensity. Furthermore, utilizing video analysis, Liu et al., (2014) leveraged frame differencing techniques to classify feeding intensity, ultimately deriving the CVFAI metric.

Alternatively, several studies have adopted indirect observational methodologies. Liu et al.,^19^ and Cao et al., (2018) assessed feeding intensity by quantifying feed consumption across the pre-, intra-, and post-feeding stages. Adopting a similar paradigm, Li et al.,^15^ quantified uneaten pellet residue utilizing an adaptive threshold technique. Based on expert inference systems, Wu et al.,^29^ and Hu et al.,^12^ formulated feeding optimization protocols, which were empirically validated through feedback loops in commercial farming environments. Despite these advancements, the majority of the aforementioned studies relied on conventional machine learning algorithms. Although effective under specific conditions^27^, these approaches are fundamentally limited by their dependence on manual feature engineering. Consequently, their classification accuracy and real-time processing capabilities remain suboptimal for deployment in large-scale, automated aquaculture.

In contrast, deep learning utilizes deep neural networks to autonomously extract hierarchical features across multiple dimensions. Unlike traditional machine learning, it bypasses the need for manual feature engineering by inherently learning richer, more complex representations. Deep learning has demonstrated remarkable efficacy across diverse domains, including agriculture and healthcare, and has been extensively applied in aquaculture for tasks such as species classification and live fish recognition.

Concurrently, significant advancements have been made in the application of deep learning to analyze fish feeding behavior. Early adoptions, such as the optimized LeNet-5 architecture proposed by Zhou et al.,^36^, achieved a detection accuracy of 90%. Han,^9^ subsequently introduced a method combining CNNs with spatiotemporal data to effectively classify various schooling behaviors. Advancing structural efficiency, Feng et al.,^8^ integrated a 3DGlore module into a ResNet backbone, developing a lightweight recognition algorithm that attained 92.68% accuracy. Expanding beyond visual data, Du et al.,^6^ and Zeng et al.,^31^ quantified foraging behavior using acoustic signals, employing a MobileNetV3-SBSC network with Mel-spectrograms and an improved Swin Transformer, respectively.

More recent studies have further refined these multimodal and hybrid architectures. Wu,^30^ evaluated feeding intensity by applying Gray-Level Co-occurrence Matrix (GLCM) and Gray-Level Gradient Co-occurrence Matrix (GLGCM) analyses to surface splashes. Highlighting the efficacy of hybrid networks, L. Xu et al., (2024) augmented the lightweight MobileViT architecture with a Convolutional Block Attention Module (CBAM) and a Bidirectional Long Short-Term Memory (BiLSTM) network, achieving 98.61% accuracy in recognition tasks. Huang,^13^ assessed Nile tilapia (Oreochromis niloticus) feeding using an enhanced VGG16 model applied to audio spectrograms, while Liu et al.,^20^ fused an attention-based ResNet with ConvNeXt for behavioral analysis. Furthermore, Zhang et al.,^33^ introduced FFishNet-YOLOv8 to detect active feeding via open-mouth identification, and Wu et al.,^28^ proposed STCA-MobileViTv3, a spatiotemporal co-attention network optimized for underwater video analysis. Collectively, these studies demonstrate that deep learning frameworks consistently surpass conventional analytical techniques in detection accuracy and operational robustness.

Despite recent advancements, the majority of existing studies rely on simulated, ideal lighting conditions within laboratory settings. Consequently, these models often fail to generalize to the high turbidity and splash interference characteristic of commercial aquaculture environments. Because controlled settings cannot adequately replicate the dynamic complexities of real-world production, robust feeding detection within practical RAS necessitates further investigation.

Addressing the limitations of existing methods, this study proposes a hybrid vision-based framework (HVIT) for accurate quantification of fish feeding intensity in industrial RAS, where high-density populations, water surface disturbances, and dynamic group behavior pose significant challenges to visual analysis. Unlike generic hybrid deep learning models, the HVIT architecture is explicitly designed to decouple these specific environmental interferences. By employing a parallel extraction strategy, the CNN is constrained to isolate high-frequency local textures (e.g., intense and localized surface splashing), while the ViT simultaneously models the low-frequency global context (e.g., broader schooling aggregation patterns). Furthermore, the incorporated Long Short-Term Memory (LSTM) module is explicitly calibrated to process these fused spatiotemporal features over empirically defined intervals, thereby capturing the complete dynamic lifecycle of a feeding event from initiation to quiescence within a noisy RAS environment.

Specifically, the primary contributions of this study are summarized as follows:

1.Application-Specific Integration: We integrated existing CNN, Vision Transformer, and LSTM components into a parallel dual-stream pipeline explicitly tailored for aquaculture. This integration leverages the complementary strengths of local texture extraction and global context representation to effectively mitigate severe visual degradation caused by real-world water surface reflections.
2.Dynamic Spatiotemporal Adaptation: The framework adapts established static image analysis techniques into a dynamic behavioral tracking system by explicitly constructing temporal sequences (T = 5, spanning 15 s) suitable for capturing the physical continuum of fish feeding behavior in industrial RAS.
3.Industrial Empirical Evaluation: A major contribution of this work is the rigorous empirical validation of this hybrid deep learning approach under realistic, high-density, and noisy production conditions. Validated exclusively on a real-world commercial RAS dataset using video-level cross-validation, this study provides highly reliable evidence and practical baselines for deploying complex AI integrations in precision aquaculture.

## Materials and methods

### Materials

**Fig. 1:**
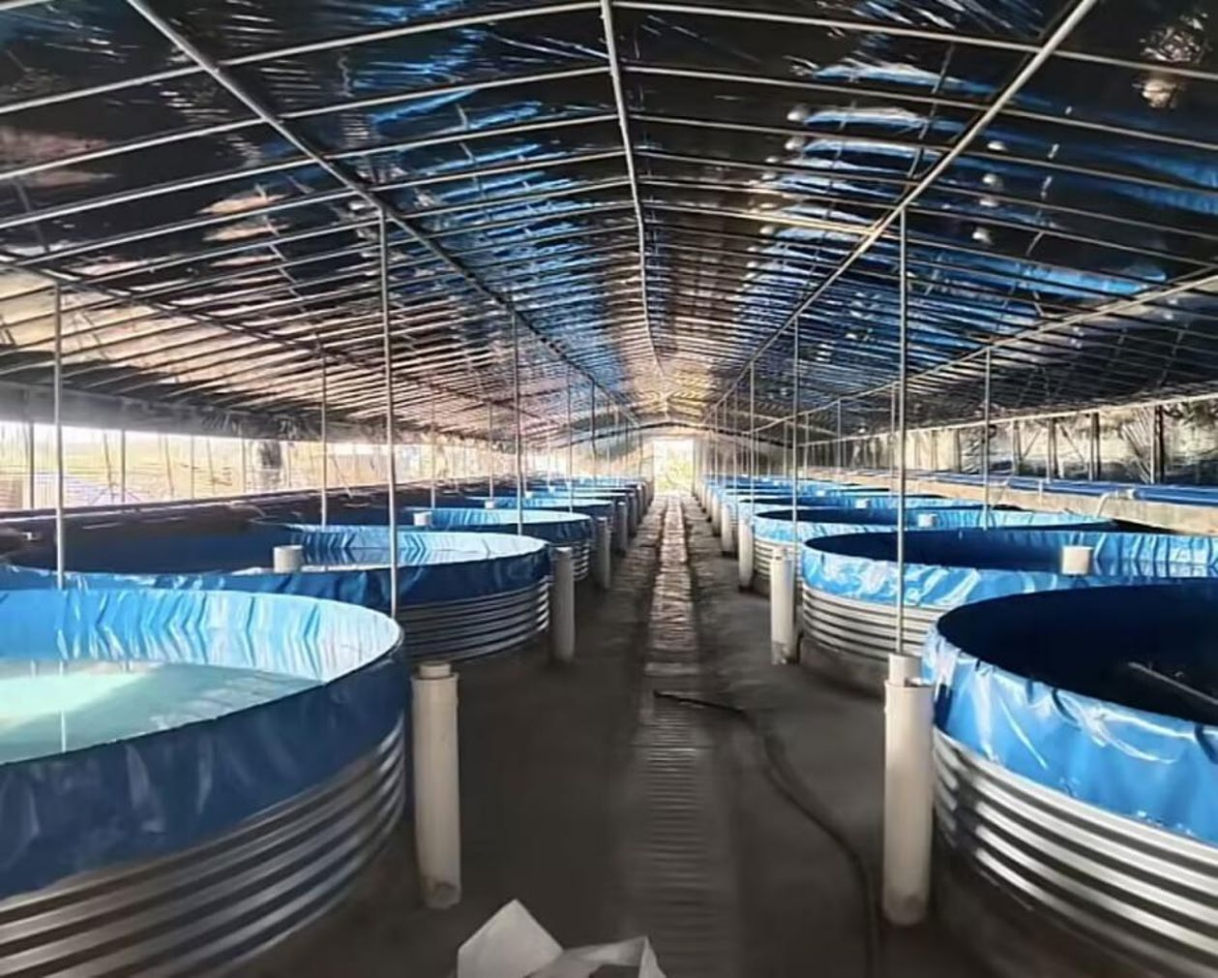
Guangxi Haoheng aquaculture base.

Data acquisition was conducted within a commercial RAS at the Haoheng Aquaculture Base in Guigang, Guangxi, China (Fig. 1). The facility specializes in the intensive cultivation of largemouth bass (Micropterus salmoides), housing a total population exceeding 20,000 individuals. The specific rearing tank utilized for this experiment measured 4.0 m in diameter and 1.5 m in height, with an operational water depth of 1.4 m. This experimental tank was stocked with 1,200 flow-conditioned (commercially termed “exercised”) largemouth bass, with an approximate market value of 14 RMB/kg. Prior to data collection, the fish had been reared in this specific tank for four months, ensuring complete acclimation to the hydrodynamic and environmental conditions of the RAS. The average body mass of the specimens was 500 g. Water quality parameters were continuously regulated, maintaining a dissolved oxygen (DO) concentration of 6.0 ± 1.0 mg/L and a temperature range of 25–27 °C. Feeding protocols were strictly scheduled twice daily at 09:00 and 17:00, utilizing an extruded commercial diet (Shandong Tongwei Feed Co., Ltd.).

### Creation of data set

**Fig. 2:**
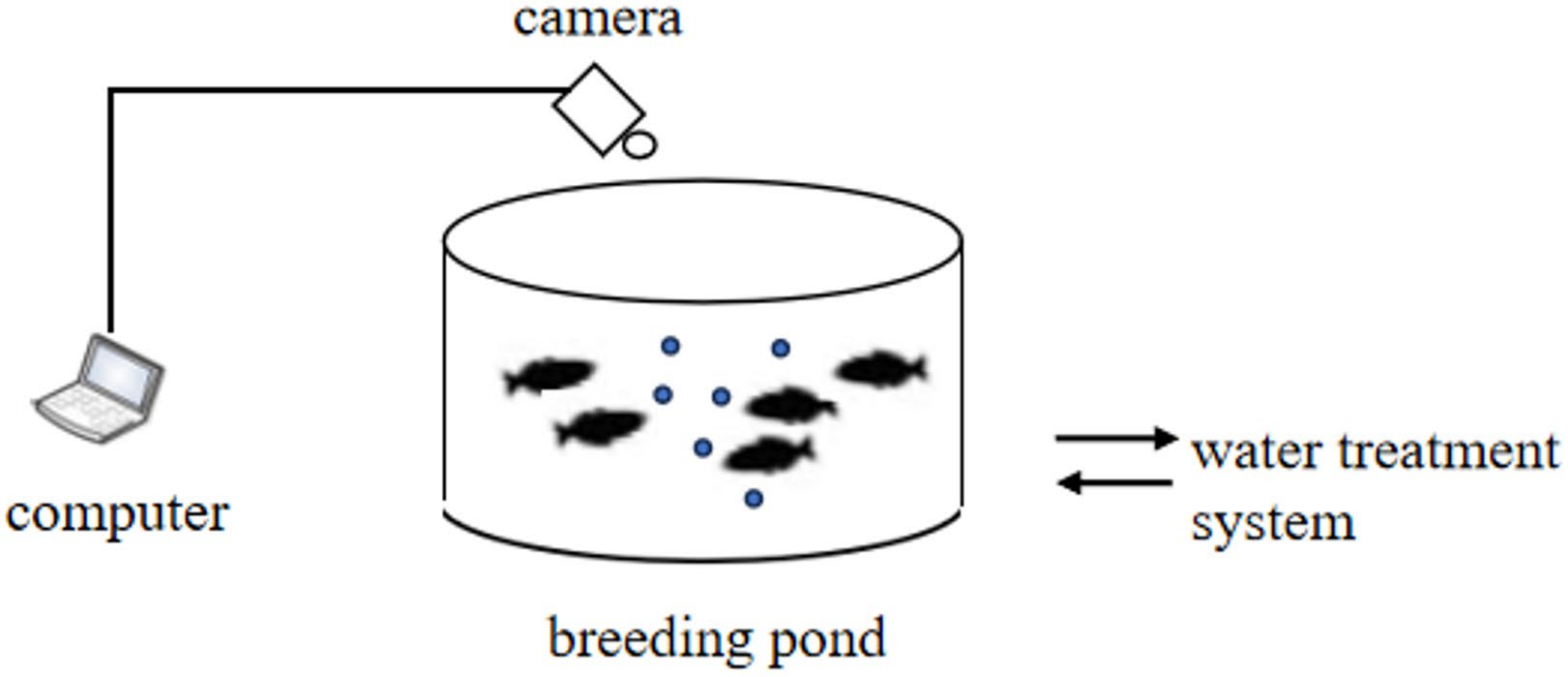
Data collection illustration.

The data acquisition setup is illustrated in Fig. 2.

A camera was mounted at an oblique angle approximately 70 cm above the water surface. Video recording commenced two minutes prior to feed delivery and terminated only after all foraging activity had ceased and the water surface had returned to a quiescent state. This rigorous protocol ensured the comprehensive capture of the fish school’s feeding dynamics across all behavioral stages. Upon acquisition, the raw video data underwent preliminary trimming to remove extraneous footage, thereby optimizing computational efficiency for subsequent analysis. This process yielded a total of 120 isolated feeding video clips. Finally, precise temporal cropping was applied to fine-tune the initial and terminal frames of each active feeding segment, ensuring the construction of a high-fidelity dataset for model training.

To ensure rigorous evaluation and prevent data leakage, the 120 raw feeding video clips were strictly partitioned at the video level into training (80%, 96 clips) and testing (20%, 24 clips) subsets prior to any frame extraction. Following this source-level separation, frames were extracted at 3-second intervals to mitigate inter-frame redundancy. Subsequent data augmentation techniques—specifically geometric transformations (flipping and mirroring) and grayscale conversion—were applied independently within the training subset. This protocol yielded a final dataset comprising 6,037 images. The class distribution across the four feeding intensity levels was relatively balanced: 1,490 (Strong), 1,459 (Medium), 1,698 (Weak), and 1,390 (None). Furthermore, to robustly verify the model’s generalization capability and mitigate selection bias, a 5-fold cross-validation strategy was implemented at the video level.

**Fig. 3:**
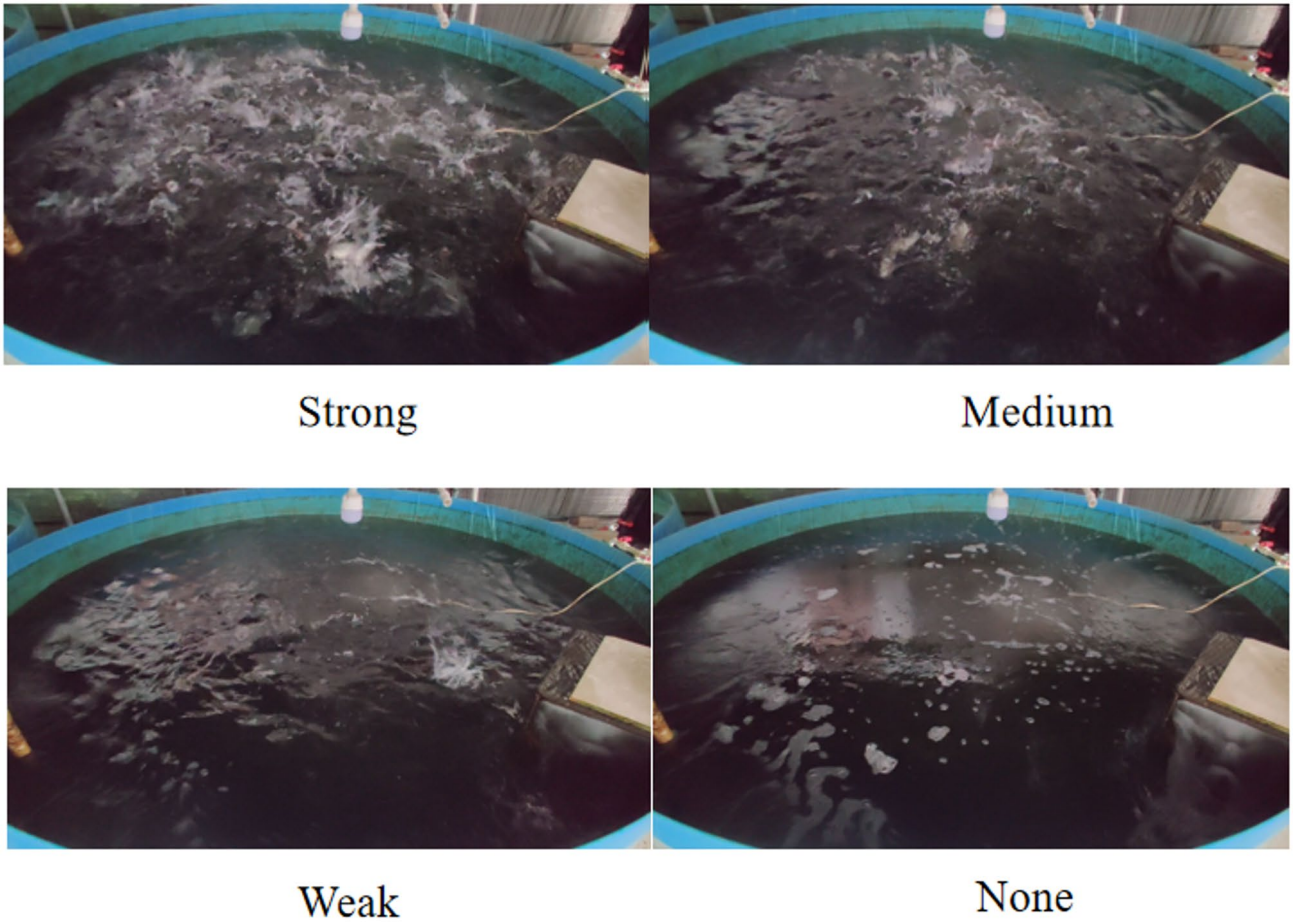
Classification of feeding intensity.

The ground-truth labeling criteria were established through a bipartite approach. First, they were grounded in established classification taxonomies from existing literature (Overli et al., 2006;^36^. Second, these criteria were empirically calibrated using the longitudinal domain expertise of aquaculture specialists at the Haoheng Aquaculture Base. To guarantee the reliability and consistency of the applied labels and minimize subjective bias, a rigorous multi-expert annotation protocol was subsequently implemented. Specifically, all video segments were independently evaluated by two experienced aquaculture researchers based on the aforementioned calibrated criteria. An independent cross-verification was then conducted to assess inter-rater reliability. In instances of annotation discrepancy (e.g., at the nuanced boundary between “Weak” and “Medium” states), a third senior aquaculture expert reviewed the footage to establish the final consensus. This double-blind labeling and conflict-resolution mechanism effectively ensured the high fidelity and consistency of the final dataset.The finalized classification metrics for the RAS imagery are detailed in Table 1 and visualized in Fig. 3.

**Table 1:** Grading table of feeding behavior.

Grade	Behavior
None	The fish do not respond to the feed, the school of fish is still, and the water surface is calm.
Weak	A few fish rise to the surface to feed, creating slight splashes and ripples on the water.
Medium	Some fish splashed water while feeding.
Strong	The fish feeds aggressively, and after feeding, continues searching for food, creating a large splash.

### Analysis of fish feeding behavior

In this study, we propose HVIT, a novel deep learning architecture for detecting fish feeding intensity that integrates ViT^5^ with CNNs. Designed specifically for industrially farmed largemouth bass, HVIT synergizes the global attention mechanisms of Transformers^26^ with the local feature extraction capabilities of CNNs. To process sequential behavioral data, Long Short-Term Memory (LSTM) networks are employed to capture temporal dynamics, complemented by an optimized activation function. Furthermore, the field-acquired dataset was augmented using spatial transformations and noise perturbation to enrich feature representation and prevent overfitting. Finally, extensive ablation studies—evaluating convolutional module selection, activation function optimization, and Transformer depth—were conducted to maximize classification accuracy and achieve optimal model performance.

#### VIT

By leveraging self-attention mechanisms to model long-range global dependencies among image patches, the ViT circumvents the reliance on traditional CNN architectures, significantly advancing performance in image classification. Its success underscores the versatility of Transformers within computer vision, catalyzing a paradigm shift toward attention-based visual models.

However, the original Transformer was engineered for one-dimensional sequential data, presenting a structural mismatch with the inherent three-dimensional topology of images (width, height, and color channels). To bridge this dimensional gap, ViT introduces a patch embedding layer. This layer divides the image into fixed-size patches, flattens them, and applies a linear projection, effectively translating spatial visual structures into a serialized sequence of embeddings. Consequently, this format seamlessly integrates with the Transformer’s robust sequence modeling capabilities. Furthermore, the strategic incorporation of a learnable classification token and positional encodings ensures that both holistic semantic features and spatial contexts are preserved, enabling ViT to efficiently process visual data and achieve state-of-the-art performance.

#### HVIT

Traditional CNNs often struggle to fully integrate the interactive dependencies between localized features and global contextual information. Conversely, the ViT effectively captures long-range global dependencies even within its shallower layers. While ViT excels at preserving spatial positional context, it inherently lacks the inductive bias required for fine-grained local feature extraction compared to CNNs. To address these complementary limitations, the present study proposes a novel hybrid architecture that fuses the deep feature extraction capabilities of CNNs with the global modeling advantages of ViT through a parallel configuration.

By operating convolutional layers and ViT modules in parallel, this design synergistically leverages the inductive biases of convolutions for local texture recognition alongside the Transformer’s ability to achieve a holistic semantic representation. Such dual-stream architectures have gained significant traction in industrial applications. A growing body of research (Mehta et al., 2022;^16,18,24,32^ suggests that parallel hybrid Transformers maintain an optimal equilibrium between classification accuracy and parametric complexity, thereby offering superior representation capabilities for complex visual tasks.

**Fig. 4:**
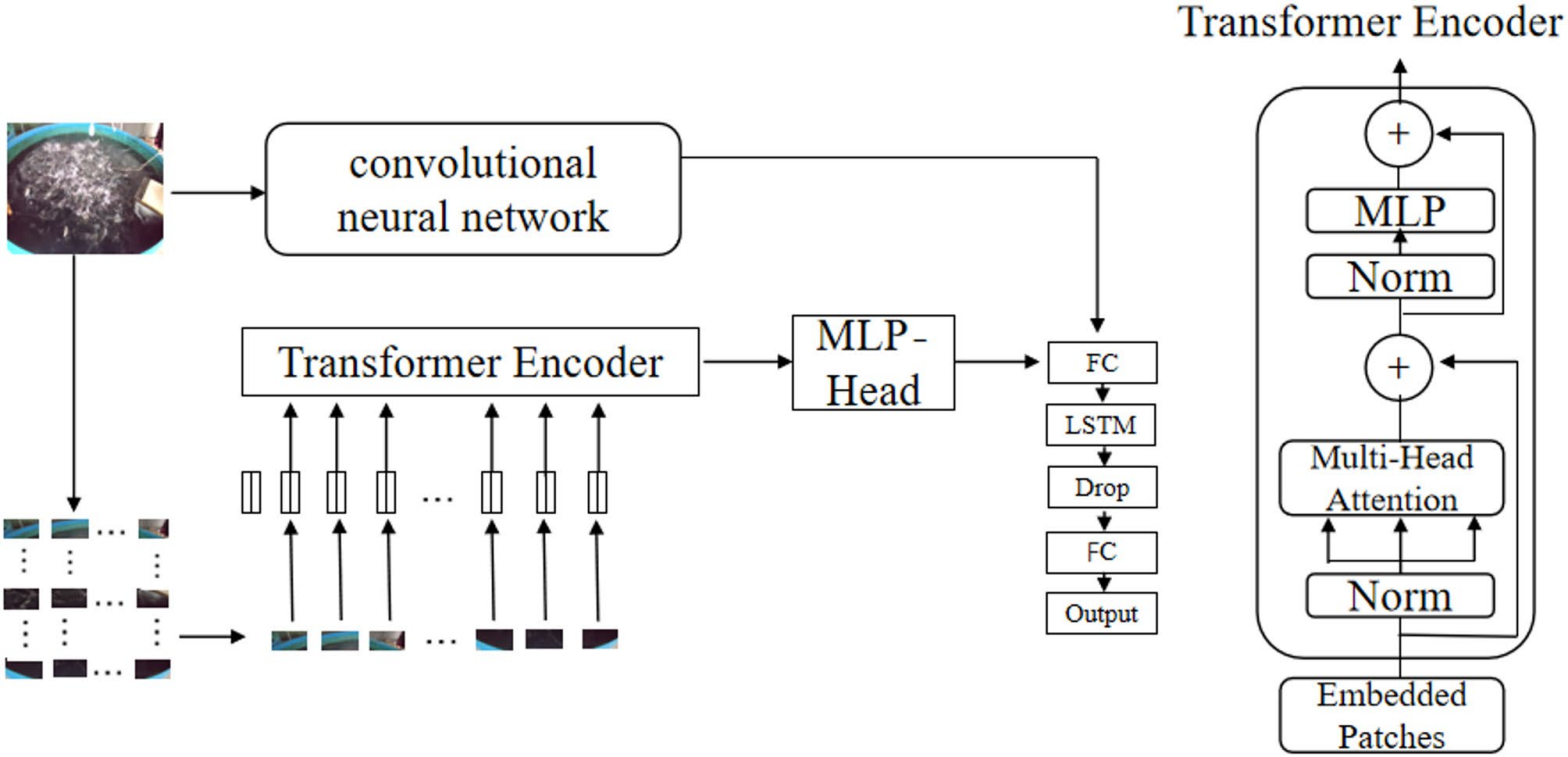
HVIT model architecture diagram.

The architecture of the proposed HVIT model is illustrated in Fig. 4. Upon entering the network, the input image is bifurcated into two parallel processing streams. The first stream directs the image through a deep CNN backbone, which employs a hierarchy of convolutional and pooling layers to progressively extract spatial features. This process reduces spatial dimensions while increasing feature map depth, effectively isolating fine-grained local information.

Simultaneously, the image is partitioned into a sequence of patches and processed in parallel by the ViT module. In this second stream, the patches are projected into a latent space via linear embedding and subsequently ingested by a Transformer encoder. This component leverages a multi-head self-attention mechanism to capture long-range global dependencies among the patches. By integrating these two streams, the model achieves a unified representation that encompasses both localized textures and global context.

Following the independent extraction paths, the high-dimensional feature maps from both streams are integrated through a feature fusion strategy. To overcome the limitations of conventional sequential architectures, our parallel fusion strategy isolates intense local noise (e.g., sudden water splashes) so it does not interfere with the ViT’s global attention mechanism. This synthesis bridges the CNN’s sensitivity to local textures with the ViT’s global contextual awareness, while simultaneously accounting for the latent temporal dependencies inherent in sequential feeding dynamics. To capture these temporal features, a Long Short-Term Memory (LSTM) layer is implemented. The fused high-dimensional spatiotemporal descriptors fed into the LSTM are explicitly structured to represent the unique temporal life cycle of a RAS feeding event—from the initial anticipatory movement to the aggressive surface splashing, and finally, the return to a quiescent state. This is followed by a Dropout layer to mitigate overfitting and enhance the model’s generalization robustness. This architecture fully leverages the complementary strengths of convolutional and attention-based modules to refine the depth of image analysis. Finally, a fully connected (FC) layer generates the prediction results to finalize the classification of fish feeding intensity.

To ensure reproducibility, the temporal sequence construction prior to the LSTM module is explicitly defined as follows: Rather than processing isolated images independently, the framework groups 5 consecutive frames (representing a continuous 15-second observation window from the same video clip) to form a single temporal sequence. Each frame in the sequence independently traverses the parallel CNN and ViT streams, generating a high-dimensional fused feature vector. These 5 consecutive feature vectors are then sequentially stacked along the temporal dimension, transforming the isolated spatial features into a continuous spatiotemporal tensor of shape (Sequence_Length, Feature_Dimension). This structured tensor is subsequently ingested by the LSTM layer, enabling the network to effectively capture and map the temporal evolution of the feeding intensity.

The architectural hyperparameters for the CNN modules were optimized through empirical testing, while the specific configurations for the LSTM and Dropout layers are as follows: The model utilizes a single-layer LSTM (depth = 1). Given that the input features have been pre-processed by the CNN and ViT branches, they represent high-dimensional spatiotemporal descriptors. To effectively model the complex temporal patterns within this high-dimensional space, the number of LSTM hidden units was set to 256. To facilitate the preservation of long-term dependencies, the forget gate bias was initialized to 0.2, utilizing a sigmoid activation function. Additionally, the Dropout rate was established at 0.3 to ensure structural regularization during training.

### Model training

To verify the effectiveness of the proposed method, we employed various training strategies to train the baseline networks. These strategies included experiments focused on the selection of the convolutional network module, the optimization of the activation function, and the depth of the Transformer architecture.

#### Selection of the convolutional network module

CNNs effectively transform fundamental local image features into more complex high-level features through their characteristic convolution and pooling operations, significantly advancing image recognition technology. However, the pooling process often sacrifices critical detail information, leading to the neglect of the connection between local details and the overall context. In contrast, the Vision Transformer (ViT) can grasp global information from shallower layers and outperforms CNNs in the ability to retain positional information, although it may underperform in capturing initial local details. The proposed parallel model aims to converge the local feature recognition advantages of CNNs with the global perception capabilities of ViT. By combining convolutional layers and ViT modules in a parallel manner, the architecture allows for the simultaneous consideration and utilization of both advantages during feature extraction, achieving deep mining and comprehensive analysis of image features. Through the sophisticated integration of local details and macro information, a more precise and comprehensive representation of image features is achieved, thereby enhancing the model’s performance in complex image processing tasks.

This experimental study investigates the impact on model performance by pairing a single-layer ViT in parallel with several classic network architectures, specifically ResNet18 (He K et al., 2016), VGG16 (Simonyan K et al., 2015), DenseNet121 (Huang G et al., 2017), and MobileNetV3 (Howard A et al., 2019).

#### Optimize activation function

The Mish activation function (Misra, 2020) maintains a slight negative gradient for non-positive inputs, resulting in a continuously differentiable, smooth profile. This smooth landscape facilitates consistent gradient flow, particularly in deep architectures where Mish has demonstrated a distinct performance advantage over the traditional Rectified Linear Unit (ReLU) (Nair et al., 2010).

When evaluating activation functions, performance across an infinite interval is critical to circumventing gradient saturation, which can otherwise impede convergence velocity. A lower-bounded curve is advantageous due to its inherent regularization effects. Furthermore, the non-monotonic nature of Mish ensures that specific negative inputs yield negative outputs, thereby enhancing the model’s non-linear expressiveness and optimizing gradient propagation. Unlike ReLU, which suffers from the “dying ReLU” problem due to its zero-gradient identity for negative values, Mish leverages its mathematical continuity to mitigate gradient vanishing. This continuity is instrumental in refining the model’s recognition capabilities and ensuring stable gradient properties throughout training.

Consequently, this study systematically evaluates the impacts of ReLU and Mish on model performance within the same architectural framework to identify the optimal configuration for feeding intensity detection.

#### Transformer in-depth experiment

Ablation experiments were conducted to systematically investigate the impact of Transformer depth on model performance. The network depth was evaluated at discrete intervals of 0, 1, 3, 5, and 7 layers. A depth of 0 denotes a configuration consisting exclusively of the ResNet18 backbone without the ViT component, effectively serving as a pure CNN baseline. This configuration functions as the control group, while the subsequent depths represent the experimental hybrid architectures.

To optimize the model for multi-class classification, the Cross-Entropy Loss function was employed as the objective criterion. This standard metric minimizes the divergence between the predicted softmax probability distribution and the ground-truth label distribution, thereby refining the model’s internal parameters during backpropagation.

By comparing these varying depths, this study aims to elucidate how the integration of Transformer layers influences global feature acquisition and generalization robustness. Utilizing the depth-0 model as a performance baseline allows for a quantitative assessment of the marginal gains provided by the Vision Transformer. It is hypothesized that increasing Transformer depth enhances the capacity to capture complex high-order dependencies; however, this must be balanced against the risk of overfitting and increased computational overhead. Consequently, identifying the optimal depth is essential for maximizing predictive accuracy while maintaining structural efficiency.

### Evaluation method

In classification tasks, the efficacy of the proposed algorithm is quantified using a suite of standardized evaluation metrics: Accuracy, Precision, Recall, and the F1-score. Accuracy serves as a primary indicator of the model’s aggregate classification performance across all categories. In contrast, Precision and Recall provide a more granular evaluation, particularly when addressing class imbalances within the dataset—common in biological data. Precision measures the fidelity of positive predictions, while Recall (sensitivity) assesses the model’s ability to identify all relevant instances of a specific class. The F1-score represents the harmonic mean of precision and recall, providing a balanced metric for model robustness.

The mathematical definitions for these metrics, based on the components of the confusion matrix—True Positives (TP), True Negatives (TN), False Positives (FP), and False Negatives (FN)—are as follows:

$$\:\begin{array}{c}Accuracy\:=\:\frac{\mathrm{T}\mathrm{P}+\mathrm{T}\mathrm{N}}{\mathrm{T}\mathrm{P}+\mathrm{T}\mathrm{N}+\mathrm{F}\mathrm{P}+\mathrm{F}\mathrm{N}}\times\:100\%\:\#\left(1\right)\end{array}$$

$$\:\begin{array}{c}Precision\:=\:\frac{\mathrm{T}\mathrm{P}}{\mathrm{T}\mathrm{P}+\mathrm{F}\mathrm{P}}\times\:100\%\#\left(2\right)\end{array}$$

$$\:\begin{array}{c}Recall\:=\:\frac{\mathrm{T}\mathrm{P}}{\mathrm{T}\mathrm{P}+\mathrm{F}\mathrm{N}}\times\:100\%\#\left(3\right)\end{array}$$

$$\:\begin{array}{c}F1-Score\:=\:\frac{2\times\:\mathrm{R}\mathrm{e}\mathrm{c}\mathrm{a}\mathrm{l}\mathrm{l}\times\:\mathrm{P}\mathrm{r}\mathrm{e}\mathrm{c}\mathrm{i}\mathrm{s}\mathrm{i}\mathrm{o}\mathrm{n}}{\mathrm{R}\mathrm{e}\mathrm{c}\mathrm{a}\mathrm{l}\mathrm{l}+\mathrm{P}\mathrm{r}\mathrm{e}\mathrm{c}\mathrm{i}\mathrm{s}\mathrm{i}\mathrm{o}\mathrm{n}}\times\:100\%\#\left(4\right)\end{array}$$

## Results and discussion

### Training details

Experiments were executed on the AutoDL cloud computing platform. The software stack leveraged the PyTorch deep learning framework (version 1.8.0) integrated with CUDA 11.1.1 for GPU-accelerated parallel computing. Python (version 3.8) served as the primary programming language, ensuring a high-performance and scalable development environment. The operating system utilized was Linux (Ubuntu 18.04.5 LTS), and the hardware infrastructure comprised an Intel Xeon Gold CPU paired with an NVIDIA GeForce RTX 3060 Ti GPU (8GB VRAM).

Prior to model training, the network hyperparameters were initialized as follows: input image dimensions were standardized to 512$\:\times\:$512 pixels; the initial learning rate was established at 0.001 with a weight decay of 0.0001 to prevent overfitting; the batch size was set to 16, and the momentum coefficient was configured at 0.937 to accelerate convergence during stochastic gradient descent (Table 2).

**Table 2:** Hyperparameter settings for this experiment:

Training parameters	Parameter value
Image size	512 × 512
Batchsize	16
Initial learning rate	0.001
weight decay	0.0001
Momentum	0.937

### Prediction results

#### Experimental results and analysis of CNN module selection

**Fig. 5:**
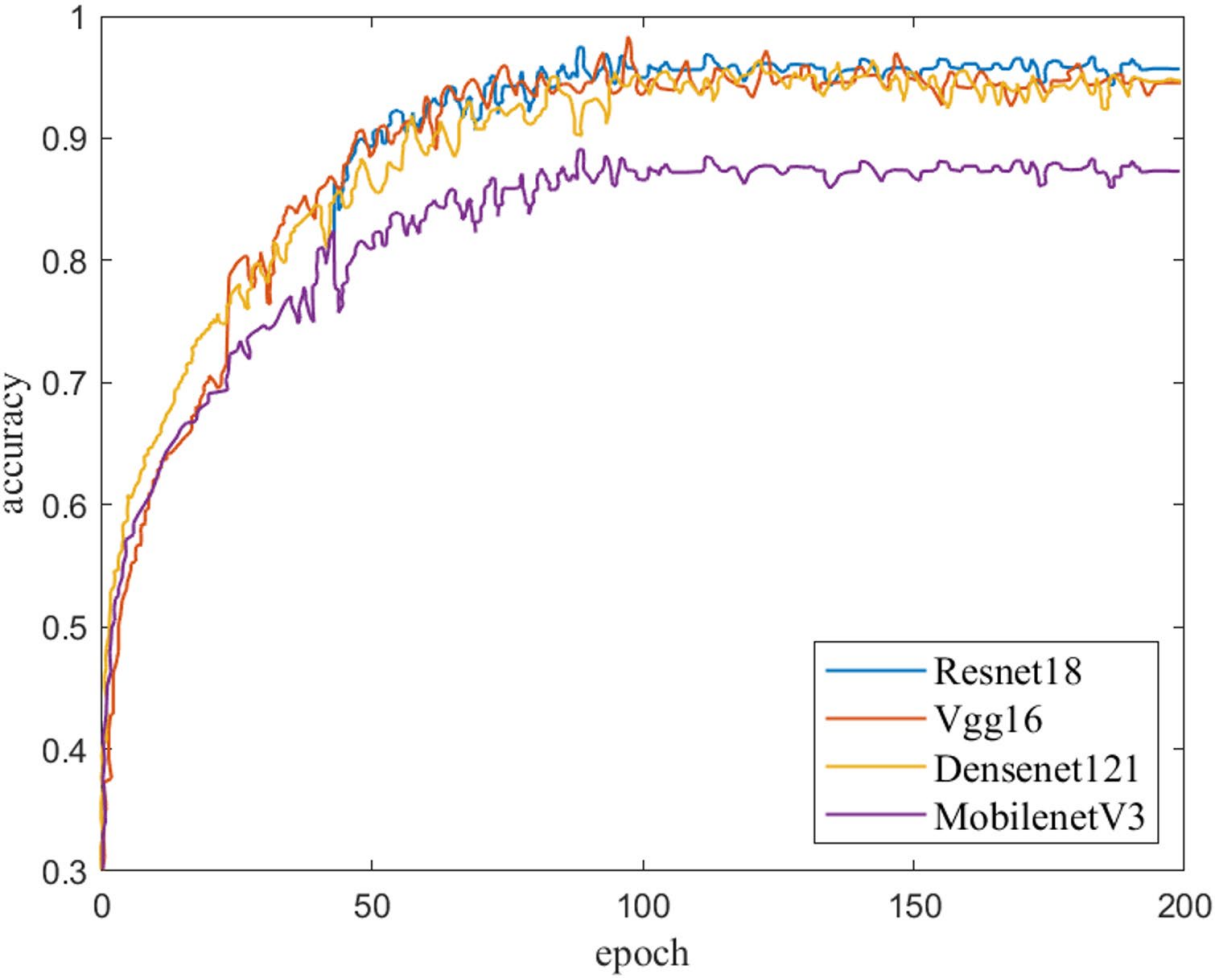
Accuracy curve of convolutional network selection experiment.

This study investigates the impact of several classical CNN models—ResNet18, VGG16, DenseNet121, and MobileNetV3—combined in parallel with a single-layer Vision Transformer (ViT) on overall model performance. The experimental results are presented in Table 3; Fig. 5.

**Table 3:** Comparison of Experimental Results for Convolutional Networks.

Model	Precision (%)	Accuracy (%)	Recall (%)	F1-score
Resnet18	96.70	96.67	96.76	0.96
Vgg16	93.58	93.55	93.63	0.93
Densenet121	92.69	92.67	92.80	0.92
MobilenetV3	87.22	87.18	87.28	0.87

In this study, several classical CNN architectures were comprehensively compared to investigate their adaptability to the task. The networks evaluated include ResNet18, VGG16, DenseNet121, and MobileNetV3, each combined with a parallel architecture incorporating a single-layer Vision Transformer (ViT). The objective was to analyze the performance of these architectures in the target task and subsequently select the optimal model for further in-depth investigation.

The dataset of fish school feeding intensity constructed in this study was first preprocessed and then divided into training and testing sets at a ratio of 8:2. During training, the model performance was evaluated using several metrics, including accuracy, precision, recall, and F1-score. As shown in Tables 3, 4, the ResNet18 model outperformed the other models across all evaluation metrics, demonstrating superior performance. Specifically, ResNet18 achieved an accuracy of 96.67%, which is substantially higher than that of the other models. Similarly, its precision, recall, and F1-score reached 96.70%, 96.76%, and 0.96, respectively, indicating strong feature extraction capability and good generalization performance.

Further examination of the accuracy curves in Fig. 5 shows that the performance of ResNet18 exhibits a steady upward trend as training progresses, without evident signs of overfitting. This observation indicates the robustness of the ResNet18 model during training and reflects its effectiveness in mitigating the vanishing gradient problem. Although VGG16 and DenseNet121 demonstrate slightly lower performance, they still exhibit strong learning capabilities. In contrast, MobileNetV3 performs relatively poorly in this experiment, which may be attributed to its relatively small number of parameters and limited computational capacity.

Based on the experimental results, and considering accuracy, model stability, and training efficiency, ResNet18 was selected as the convolutional neural network architecture for subsequent research. This choice is primarily supported by its superior performance and its effectiveness in addressing the vanishing gradient issue in deep networks. Future work will focus on further optimizing the ResNet18-based architecture and exploring additional approaches to improve the accuracy of fish school feeding intensity detection.

#### Experimental results and analysis of optimized activation function

The original activation function used in the ResNet18 network is the Rectified Linear Unit (ReLU). The primary advantages of this activation function lie in its simple and computationally efficient formulation, as well as its effectiveness in mitigating the vanishing gradient problem, both of which facilitate the training of deep learning models. Its concise mathematical form results in relatively low computational cost during both forward and backward propagation, thereby accelerating the training process.

However, ReLU has a notable limitation: the function outputs zero for all negative inputs, which leads to zero gradients in this region. Under such conditions, if the activation value of a neuron falls within this range, it will not contribute to weight updates during backpropagation. Consequently, the neuron becomes unresponsive to variations in input data, a phenomenon commonly referred to as the “dying ReLU” problem. This issue can reduce the learning capacity and representational power of the model, as such inactive neurons remain non-responsive throughout the training process.

To address this limitation, Diganta Misra introduced Mish, a novel nonlinear activation function specifically designed for deep learning tasks. A series of experiments have demonstrated its effectiveness, highlighting its potential to improve the performance of deep neural networks.

The formula for the Mish activation function is:

$$\:\begin{array}{c}Mish\left(\mathrm{x}\right)=x\ast\:\mathrm{tanh}\left(\mathrm{ln}\left(1+{\mathrm{e}}^{\mathrm{x}}\right)\right)\#\left(5\right)\end{array}$$

Compared with the ReLU activation function, the Mish activation function maintains a small negative gradient in the non-positive input region, resulting in a smoother functional curve. This slight negative gradient improves gradient flow and facilitates effective information propagation in deep neural networks. Consequently, when the number of convolutional layers increases, models employing the Mish activation function often achieve higher accuracy than those using ReLU.

**Fig. 6:**
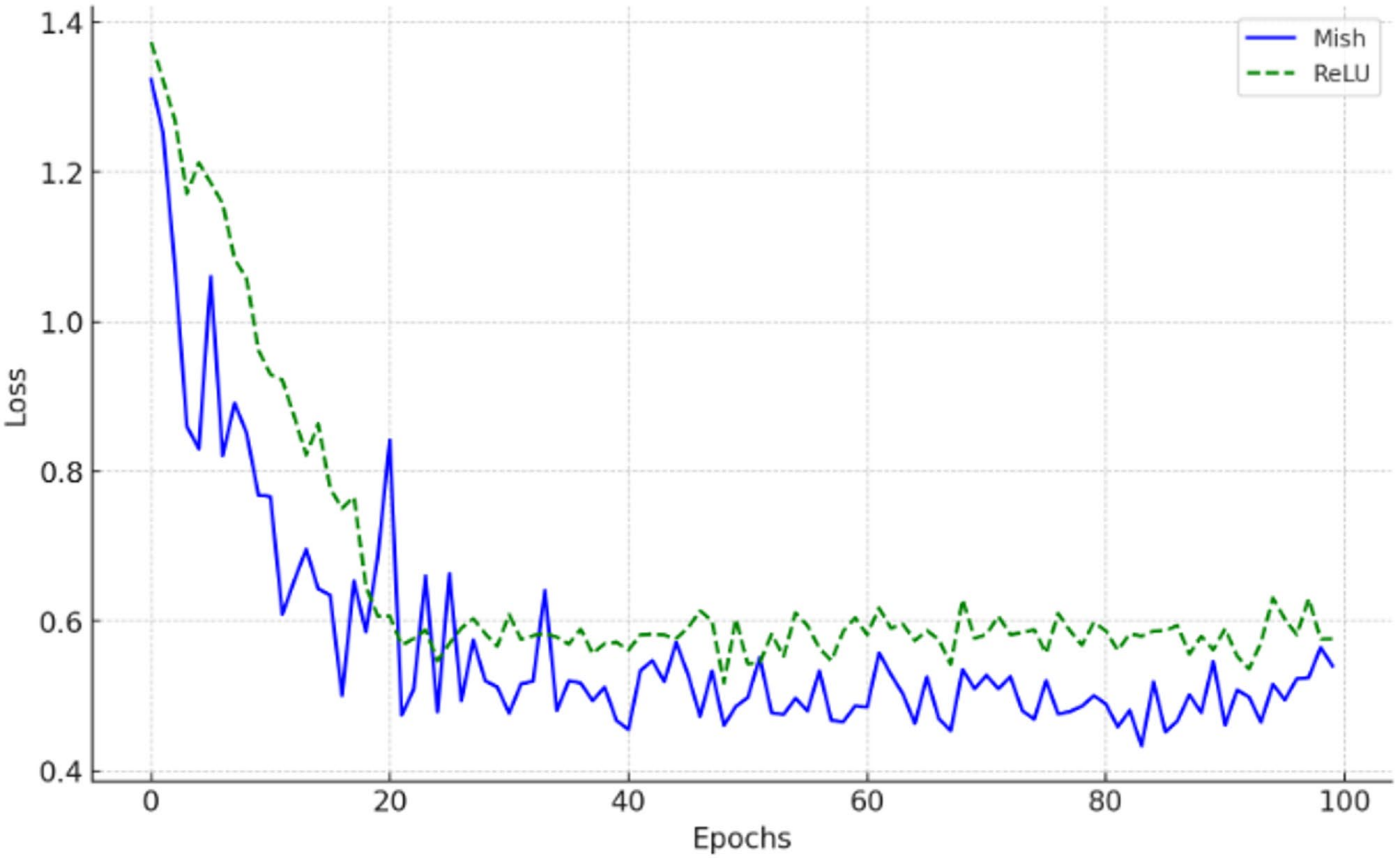
Loss curve.

This study aims to evaluate the specific impact of two activation functions—ReLU and Mish—on model performance under the same model framework. The dataset used in the experiment is described in detail in Sect.  3.1 and consists of images representing different feeding intensity levels. Among them, 80% of the images were used as the training set, while the remaining 20% were used for model validation. All parameters were kept unchanged except for the activation function, and the models were trained for 100 epochs. The corresponding loss curves are shown in Fig. 6.

The experimental results are illustrated in Fig. 6, showing the variation of the loss rate across training epochs. It is clearly observed that while the model employing the ReLU activation function converges faster during the initial stages of training, the model using the Mish activation function achieves a lower loss rate as training progresses. Particularly after 35 epochs, the Mish-based model not only converges stably but also maintains a consistently lower loss rate than the ReLU-based model. Although ReLU is widely adopted in practice due to its computational simplicity, the Mish activation function proves to be a superior choice for fish feeding intensity detection. Due to its self-regularizing properties, Mish provides better training stability and facilitates continuous learning and performance improvement over extended training durations—advantages that ReLU lacks. Therefore, in the subsequent experiments of this study, Mish was selected as the model’s activation function to achieve superior performance. This decision, based on empirical observations of the loss rate, aims to enhance the model’s long-term learning capacity and its generalization capability on unseen data.

#### Experimental results and analysis of transformer depth

This experiment aims to investigate the specific impact of varying Transformer depths on model performance. The Adam optimizer was selected for the experiment, with an initial learning rate set at 0.001. A StepLR learning rate scheduling strategy was adopted, where the learning rate was multiplied by a factor of 0.1 every 10 training epochs. Based on this configuration, training and validation were conducted on five network models with Transformer depths set to 0, 1, 3, 5, and 7, respectively. The resulting accuracy and loss rates are summarized in Table 4.

**Table 4:** Accuracy and Loss Rate of the Model.

Model	Accuracy (%)	Loss (%)
Depth = 0	95.57	8.2
Depth = 1	97.32	8.0
Depth = 3	97.85	7.7
Depth = 5	98.93	7.1
Depth = 7	98.83	7.5

The experimental results indicate that model performance does not improve linearly with the increase in Transformer depth. When the Transformer depth is 0, the model consists exclusively of the convolutional network component, serving as the control group for this experiment. As the Transformer depth increases, a gradual upward trend in model performance is observed. Specifically, the model reaches its peak performance at a depth of 5, achieving an accuracy of 98.93%, which is 3.36% points higher than that of the control group (depth 0). This finding suggests that appropriately increasing the Transformer depth can effectively enhance the classification accuracy of the model. However, when the depth is further increased to 7, the model performance begins to decline.

This phenomenon indicates that, for the specific task of fish school feeding intensity recognition, a convolutional neural network model integrated with a Vision Transformer is feasible. Moreover, when the Transformer depth is set to five layers, the model achieves optimal performance, surpassing the model based solely on a convolutional neural network. This improvement can be attributed to the ability of an appropriately configured Transformer depth to capture richer features and facilitate effective feature fusion, thereby enhancing the model’s recognition capability. However, an excessively deep Transformer may introduce overfitting or increase the difficulty of model training, which can ultimately lead to performance degradation.

**Fig. 7:**
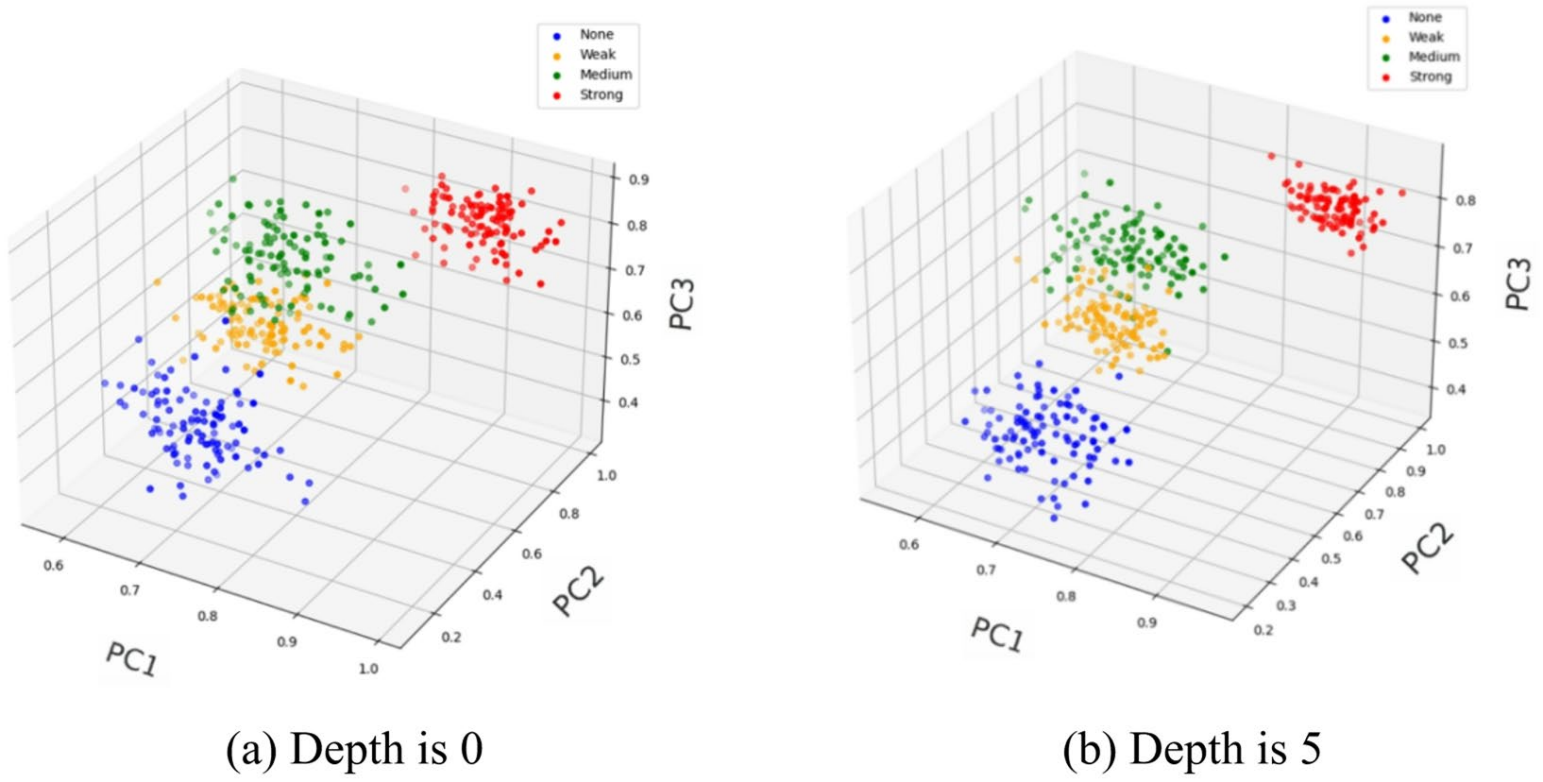
3D feature space visualization using Principal Component Analysis (PCA) to illustrate the clustering of four feeding intensity levels.

Therefore, the experimental results highlight the importance of selecting an appropriate model depth when designing deep learning architectures, ensuring that high performance is achieved while avoiding unnecessary computational overhead. To visually illustrate the model’s capability to separate different classes in the data space when the Transformer depth is set to 0 and 5, respectively, Fig. 7 presents a comparison of the classification performance of the two models on the dataset using three-dimensional scatter plots.

Figure 7a presents the 3D feature spatial distribution derived from the baseline model (Transformer depth = 0) using PCA dimensionality reduction. As observed, while the four categories are broadly distinguishable, there is substantial boundary overlap, particularly between the ‘Weak’ (orange) and ‘Medium’ (green) feeding states. This indicates that the purely convolutional architecture struggles to extract highly discriminative features for adjacent intensity levels.

In contrast, Fig. 7b illustrates the feature space mapped by the optimal HVIT framework (Transformer depth = 5). The quantitative improvement in the representation is visually evident: the intra-class variance is significantly compressed, resulting in denser, tighter clusters. Furthermore, the inter-class margins, especially the Euclidean distance separating the ‘None’, ‘Weak’, and ‘Medium’ clusters, are distinctly enlarged. This explicit spatial separation confirms that the integration of the 5-layer ViT successfully captures global semantic dependencies, thereby generating a more robust and highly discriminative latent space for complex behavioral classification.

#### Ablation experiment results and analysis

To verify the effectiveness of the proposed parallel network architecture and to validate the contributions of the LSTM layer and Dropout, multiple sets of ablation studies were conducted under the premise of consistent parameters. The experimental results are presented in Table 5.

**Table 5:** Ablation Experiment.

Model	Precision (%)	Accuracy (%)	Recall (%)	F1
Resnet18	95.43	95.57	95.51	0.95
ViT	96.34	96.29	96.36	0.96
ViT+Resnet18	96.70	96.67	96.76	0.96
ViT+Resnet18(Mish)	97.69	97.32	97.80	0.97
ViT(5 layer)+Resnet18(Mish)	98.90	98.93	98.89	0.98
ViT(5 layer)+Resnet18(Mish)+LSTM	98.95	98.95	98.90	0.98
ViT(5 layer)+Resnet18(Mish)+LSTM+Drop	99.01	99.02	99.09	0.99

As shown in Table 5, constructing a parallel architecture by integrating ResNet18 with a ViT effectively enhances the model’s feature extraction capability. Compared with the original ViT model, the classification accuracy increases by 0.38%. Furthermore, optimizing the activation function by replacing ReLU with the Mish activation function further improves model performance, resulting in a significant enhancement in fish school feeding intensity detection. Specifically, the accuracy increases by 1.03% points compared with the baseline ViT model.

Subsequently, determining the Transformer depth as five layers yields better performance, achieving an improvement of 2.64% points over the original ViT model. Finally, the incorporation of Long Short-Term Memory (LSTM) and Dropout strengthens the model’s ability to capture temporal information while mitigating overfitting. As a result, the accuracy increases by 2.73% points relative to the original ViT network.

Through the above ablation experiments, the effectiveness of the proposed fusion architecture integrating ViT with a convolutional neural network is further validated. The results demonstrate that the fused model possesses superior detection capability and can effectively accomplish the target task, highlighting its potential and practical value for real-world applications.

#### Overall experimental results of the model

**Fig. 8:**
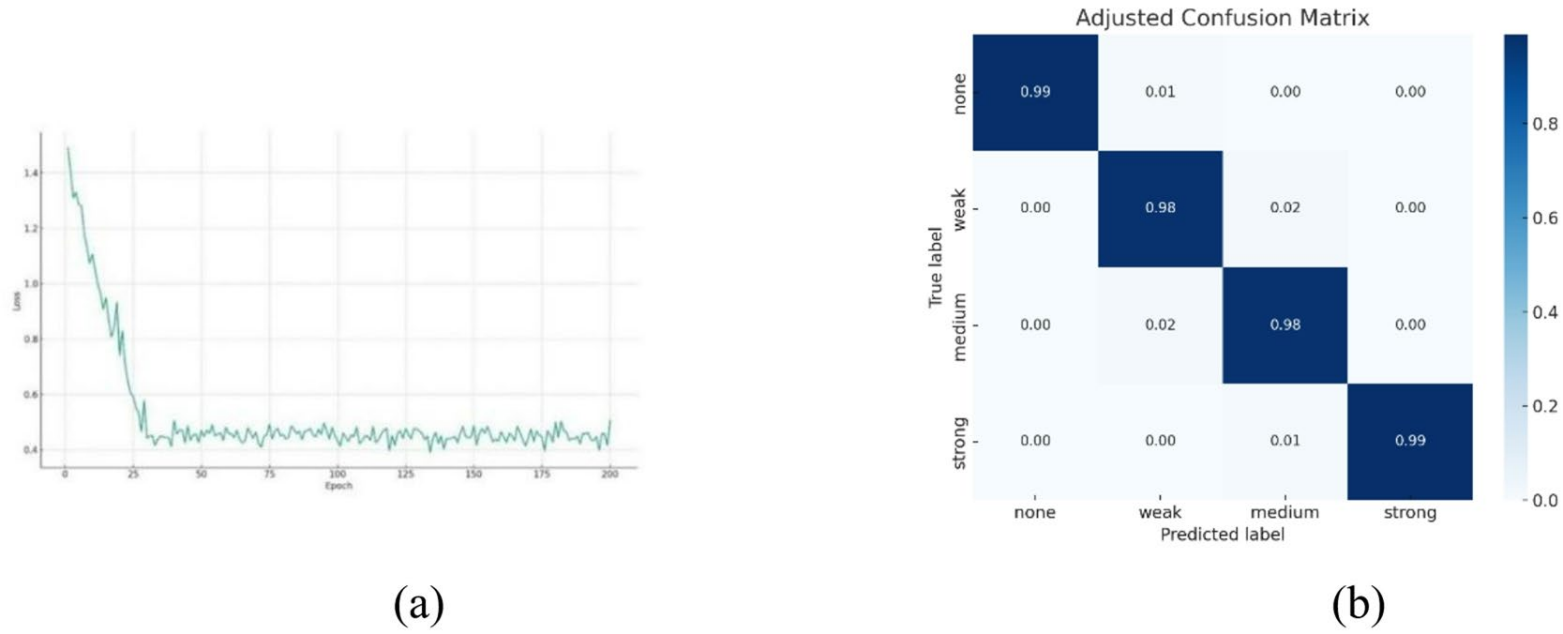
Loss curve and confusion matrix.

Figure 8a and b present the final loss curve and confusion matrix of the proposed model, respectively. (a)loss curve, (b)confusion matrix.

From Fig. 8a, it can be observed that during the early stage of training, the loss value decreases rapidly within the first 30 iterations. After this stage, the loss enters a phase of gradual oscillatory decline and eventually stabilizes at approximately 0.5. This process indicates that, after quickly adapting to the fundamental structure and patterns of the training dataset, the model begins to refine its understanding of the data and improve its predictive capability. Despite the inherent complexity of the dataset, the model is able to progressively converge to a relatively optimal state, demonstrating a good fitting ability to the provided data.

As observed in Fig. 8b, the confusion matrix demonstrates high accuracy in identifying the four levels of feeding intensity. The prediction accuracy for the “None,” “Weak,” “Medium,” and “Strong” categories all exceeds 98%. The high values along the diagonal indicate that the vast majority of predictions are accurate. The lower values off-diagonal, representing the misclassification rates, reveal minor errors in the model’s predictions. Specifically, between adjacent categories, the probability of “Weak” being misclassified as “Medium” and “Medium” as “Weak” is slightly higher than other off-diagonal values, both at 0.02. Overall, this confusion matrix indicates that the model has effectively learned the features of the data during the training process and can accurately identify different feeding intensities.

By combining the analysis of the downward trend in training loss with the confusion matrix, it can be inferred that as the loss value decreases and stabilizes, the recognition accuracy simultaneously improves, thereby achieving a robust fit to the training data. This further demonstrates that while the model adapts to the training data, it also possesses strong generalization capabilities, which are essential for fish feeding intensity recognition tasks in industrial aquaculture environments. Consequently, these findings confirm the applicability and robustness of the proposed model for identifying fish feeding intensity within industrial farming settings.

To comprehensively evaluate the robustness of the proposed HVIT framework and mitigate potential selection bias associated with a single static data split, a rigorous 5-fold cross-validation was conducted strictly at the video level. The 120 independent feeding video clips were randomly partitioned into five equal, non-overlapping subsets (24 videos per fold). In each of the five iterations, four subsets (96 videos) served as the training group, while the remaining subset (24 videos) was used for testing. Furthermore, to address potential class imbalances and penalize inter-class confusion (e.g., between ‘Weak’ and ‘Medium’ states), the Macro-averaged F1-score was incorporated alongside overall Accuracy.

**Table 6:** Results of the 5-fold cross-validation at the video level.

Fold	Testing Videos	Accuracy(%)	Precision(%)	Recall(%)	Macro F1-score
Fold 1	24	97.43	97.21	97.55	0.973
Fold 2	24	95.81	95.92	95.74	0.958
Fold 3	24	96.52	96.44	96.61	0.965
Fold 4	24	94.88	95.03	94.75	0.949
Fold 5	24	96.18	96.31	96.06	0.962
**Average ± SD**	**-**	**96.16 ± 0.94**	**96.18 ± 0.80**	**96.14 ± 1.03**	**0.961 ± 0.009**

As presented in Table 6, the HVIT model achieved an average Accuracy of 96.16% with a standard deviation of 0.92%, and a Macro F1-score of 0.961 (**±** 0.009). The narrow standard deviations across the five independent testing folds robustly demonstrate that the model maintains consistent generalization capabilities regardless of the specific data split. This confirms that the high classification performance is inherently derived from the network’s architectural efficacy in capturing spatiotemporal feeding dynamics, rather than being an artifact of a favorable dataset partition.

#### Computational efficiency and real-time feasibility

To evaluate the deployment feasibility of the proposed HVIT framework in industrial settings, it is essential to analyze its computational efficiency. The concept of “real-time” in aquaculture feeding control systems does not necessitate extremely high frame-rate processing (e.g., > 60 FPS); rather, it requires the system’s inference time to be significantly shorter than the behavioral transition periods of the fish school. Evaluated on the experimental hardware infrastructure (NVIDIA GeForce RTX 3060 Ti GPU), the optimal HVIT model (comprising the ResNet18 backbone, a 5-layer ViT, and a single-layer LSTM) contains approximately 28.6 Million parameters (Params) and exhibits a computational complexity of 124.5 GFLOPs for a full sequence forward pass. Crucially, the average inference time required to process a complete temporal sequence (i.e., a15-second observation window constructed from T = 5 high-resolution 512 × 512 frames) is merely 145 milliseconds. Given that the physical progression of feeding intensity changes typically spans tens of seconds to minutes, this 0.145-second inference speed provides an ample temporal buffer for executing mechanical actuation in automated feeders. However, it is crucial to acknowledge that this timing reflects offline inference on a high-end desktop GPU. Practical deployment in real industrial settings will inevitably introduce additional operational constraints, such as edge device hardware limitations, streaming video decoding overhead, network transmission latency, and dynamic environmental fluctuations. Therefore, rather than claiming immediate real-time deployment readiness, these results demonstrate a strong baseline of computational feasibility. Consequently, despite the hybrid architecture, the proposed model provides a highly promising technical foundation for future lightweight optimization and integration into edge-based precision aquaculture systems.

#### comparison with state-of-the-art models

To conclusively evaluate the superior performance of the proposed framework, the optimal HVIT model was benchmarked against recent State-of-the-Art (SOTA) visual recognition architectures. Specifically, we selected **ConvNeXt** (a modern, highly optimized pure convolutional network) and **Swin Transformer** (a hierarchical Vision Transformer employing shifted windows) as our advanced baselines. These models were trained and evaluated on our RAS feeding dataset under the identical 5-fold cross-validation protocol.

**Table 7:** Benchmark comparison with recent state-of-the-art architectures.

Model Architecture	Type	Accuracy (%)	Precision (%)	Recall (%)	Macro F1-score
ConvNeXt	Modern CNN	94.50 **±** 1.12	94.62	94.41	0.945
Swin Transformer	Modern ViT	95.10 **±** 1.05	95.25	95.06	0.951
**Proposed HVIT**	**-**	**96.16 ± 0.92**	**96.18**	**96.14**	**0.961**

As presented in Table 7, while both SOTA architectures demonstrate strong spatial feature extraction capabilities, the proposed HVIT framework outperforms them across all primary metrics. Specifically, HVIT achieved a 1.66% and 1.06% higher average accuracy compared to ConvNeXt and Swin Transformer, respectively. To rigorously verify that these observed gains are mathematically meaningful rather than due to random variance, paired t-tests were conducted on the 5-fold cross-validation results. The analysis confirmed that the accuracy improvements of HVIT are statistically significant (*p* < 0.05 against ConvNeXt, and *p* < 0.01 against Swin Transformer). The fundamental limitation of these static SOTA models lies in their strictly spatial analytical domain. In contrast, the HVIT framework explicitly incorporates temporal dynamics (LSTM processing over a 15-second sequence). This statistical and empirical evidence indicates that capturing the continuous behavioral evolution—rather than merely analyzing isolated static textures—is decisive for accurately quantifying dynamic feeding intensity in highly disturbed aquaculture environments.

## Conclusion

- This study presents a hybrid vision-based framework (HVIT) for accurate and robust assessment of fish feeding intensity in industrial RAS. By integrating convolutional feature extraction with global context modeling and temporal dynamics analysis, the proposed method effectively addresses the challenges of complex aquaculture environments, including high fish density, water surface disturbances, and dynamic group behavior.
- Experimental results demonstrate that the proposed framework achieves high recognition accuracy across four feeding intensity levels and outperforms conventional deep learning approaches. More importantly, the method enables reliable quantification of feeding activity, which is essential for real-time monitoring and adaptive feeding management.
- The proposed approach provides a practical technical foundation for intelligent feeding systems, demonstrating strong potential for broader application to reduce feed waste, improve feeding efficiency, and support precision aquaculture in large-scale industrial settings. Despite these promising results, this study acknowledges certain limitations that warrant further investigation. Primarily, the current empirical validation is constrained to a single commercial facility (the Haoheng Aquaculture Base) and focuses on one specific species (largemouth bass). While robust data augmentation and video-level cross-validation demonstrate internal robustness within the evaluated RAS conditions, the framework’s broad generalization capability under severe domain shifts—such as fundamentally different physical layouts, varied lighting conditions, or distinct fish species—remains unverified. Consequently, transferring this model to diverse industrial environments will naturally require future domain adaptation or fine-tuning. Therefore, future work will focus on two main trajectories: first, constructing a multi-center, cross-species external validation dataset to thoroughly evaluate domain adaptability and explore transfer learning techniques; and second, developing lightweight model variants to ensure computational feasibility for edge-device deployment in real-time feeding control systems.

## Data Availability

The data that support the findings of this study are available from the authors, but restrictions apply to the availability of these data, which were used under license from Guangxi Haoheng Aquaculture Base for the current study, and so are not publicly available. Data are, however, available from the authors upon reasonable request and with permission from Guangxi Haoheng Aquaculture Base.
